# Pulmonary Valve Replacement in an Adult Jehovah's Witness with Tetralogy of Fallot

**DOI:** 10.7759/cureus.7337

**Published:** 2020-03-20

**Authors:** Talha Ahmed, Ayesha Safdar, Sunjay Kaushal, Stacy Fisher

**Affiliations:** 1 Internal Medicine, University of Maryland Medical Center, Baltimore, USA; 2 Internal Medicine, Army Medical College, Rawalpindi, PAK; 3 Cardiothoracic Surgery, University of Maryland Medical Center Midtown Campus, Baltimore, USA; 4 Cardiology, University of Maryland Medical Center Midtown Campus, Baltimore, USA

**Keywords:** pulmonary valve replacement, adult congenital heart disease (achd), complex surgeries, jehovah's witness

## Abstract

The refusal of Jehovah's Witnesses to use blood products can limit access to cardiac surgery, as patients may not be offered surgery for complex disease, especially revision surgery. We report a successful, complex adult congenital heart disease (ACHD) surgery with intraoperative and perioperative optimization. We have tried to highlight through this case that complex ACHD surgeries can be performed in Jehovah's Witness patients with skilled perioperative and intraoperative management. The role of bovine hemoglobin in this population is being defined and was found helpful in this case.

## Introduction

The refusal of Jehovah's Witnesses to use blood products can limit access to cardiac surgery. Usually, robust perioperative and intraoperative optimization is required in order to make the surgery successful [1]. Cardiac surgery is a major surgery that still poses a risk when the option of transfusion is limited. However, when the benefit of doing surgery outweighs the risk of complications, surgery is still feasible with appropriate optimization [2]. We report a similar case of an adult Jehovah's witness who underwent a successful complex adult congenital heart disease (ACHD) surgery with perioperative and intraoperative optimization.

## Case presentation

**Figure 1:**
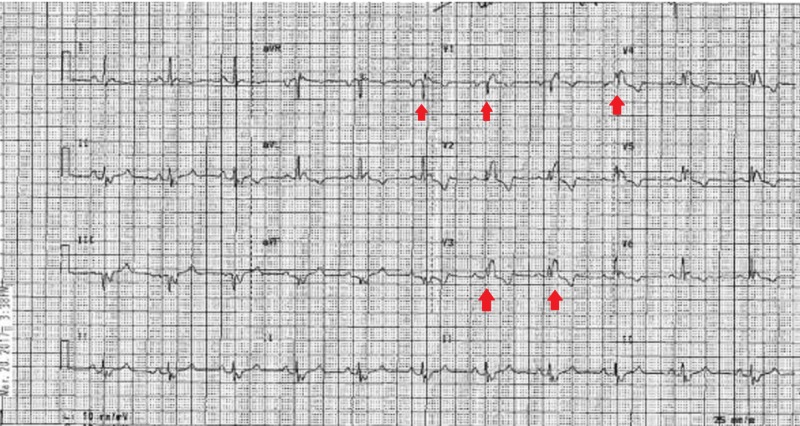
Electrocardiogram on presentation showing widened QRS complex with a right bundle branch block morphology

A 43-year-old adult Jehovah's Witness with tetralogy of Fallot and velocardiofacial syndrome had her first congenital heart surgery at the age of nine years, delayed due to blood product restriction. She presented with dyspnea and frequent palpitations. Electrocardiogram (EKG) was done, which revealed a wide QRS complex with right bundle branch block (RBBB) morphology (Figure 1).

**Figure 2:**
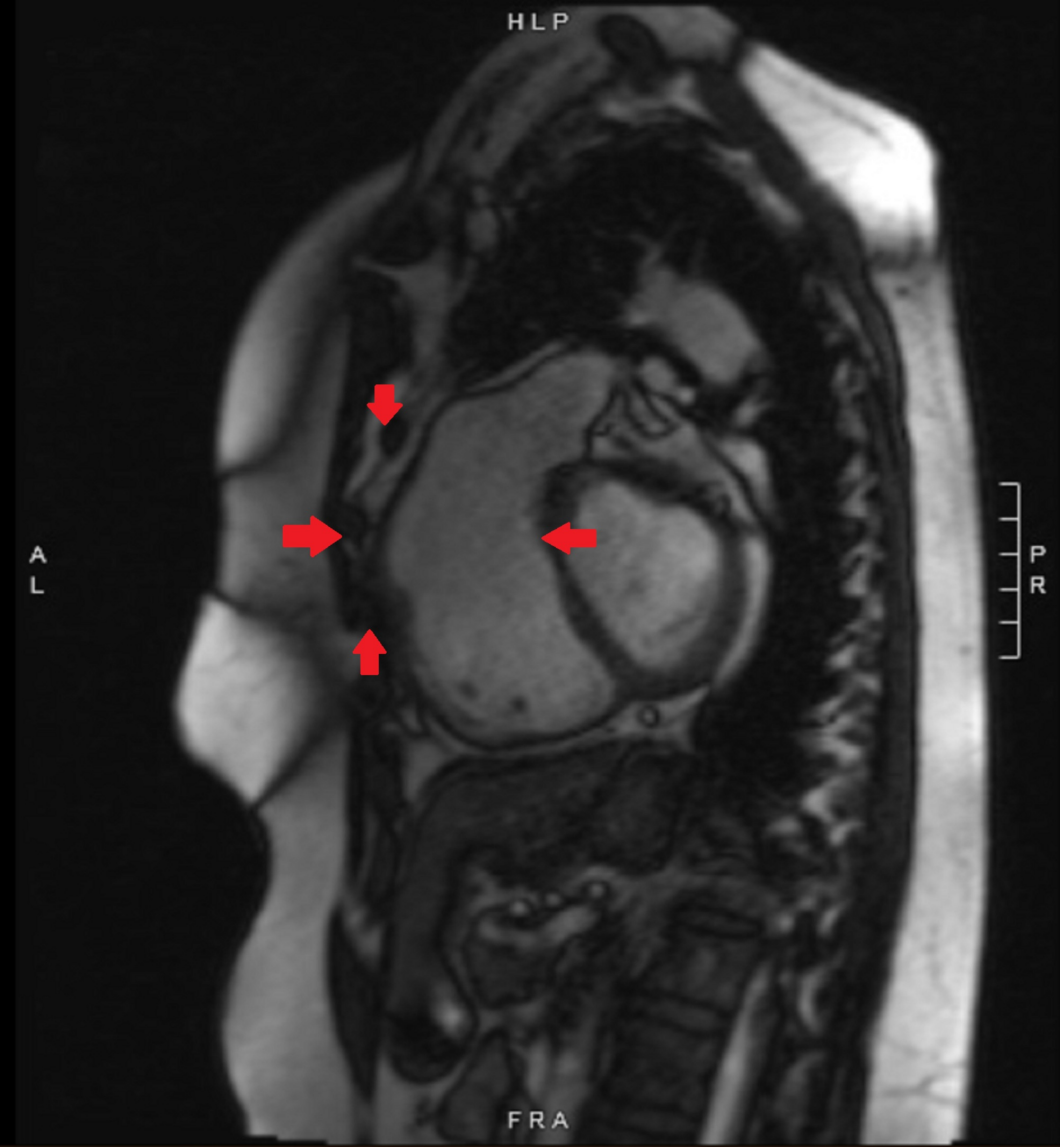
Preoperative cardiac magnetic resonance imaging showing proximity of the heart to the chest wall

**Figure 3:**
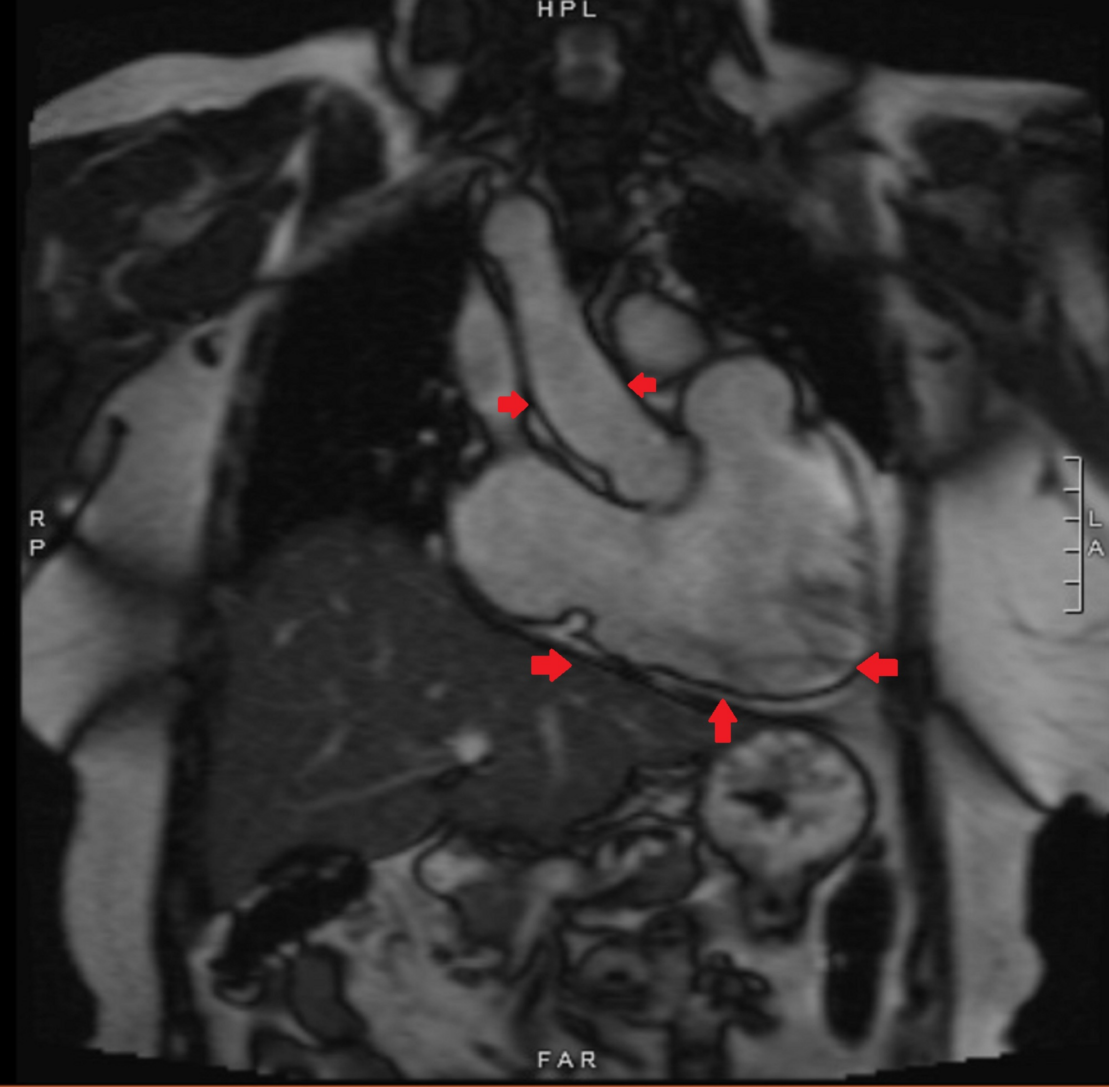
Preoperative cardiac magnetic resonance imaging showing dilated right ventricle and right ventricular outflow tract

Transthoracic echocardiogram was consistent with progressive right ventricular dilation dysfunction and normal left ventricular size and function. Magnetic resonance imaging (MRI) was performed for further elaboration of the right ventricle (RV) and the pulmonary arteries. It revealed a dilated and dysfunctional RV, pulmonic valve insufficiency with dilated main pulmonary artery, focal stenosis of the left main pulmonary artery, and adhesions of heart to the chest wall (Figures 2-3).

Surgical options included hybrid pulmonary valve repair (PVR) with a pulmonary artery (PA) band and a catheter-based stent valve versus surgical replacement. Chest anatomy and adhesions elaborated by imaging eventually made us decide on surgical pulmonary valve replacement (PVR). Pre and postoperative erythropoietin were given. Congenital heart surgeons used cell saver technology, colloids, and bovine hemoglobin. The patient had a surgical PVR and bovine pericardial patch augmentation of the left pulmonary artery, which also secured the valve anteriorly. Time on cardiac bypass was minimized and heparin used intraoperatively was reversed using protamine.

## Discussion

Patients who are Jehovah's Witnesses pose difficult ethical and moral dilemmas for surgeons because of their refusal to receive blood and blood products. The reluctance to use whole blood and its main fractions (both by Jehovah's Witnesses and on rare occasions when other individuals prefer to avoid blood transfusions) has spurred the development of perioperative and intraoperative protocols to improve 'patients' outcomes particularly after complex surgeries including cardiothoracic surgeries [3,4]. Products that are generally acceptable include recombinant erythropoietin, recombinant factor VIIa, and artificial blood substitutes, whereas those that may be acceptable include platelet fractions, albumin, immunoglobulins, cryoprecipitate, interferons, and red cell fractions including human hemoglobin [5]. 

Preoperative optimization of patients who are Jehovah's Witnesses (especially those with anemia) may be achieved using intravenous iron infusions and erythropoietin-stimulating agents to augment erythropoiesis as was done in our patient [6]. Erythropoiesis is a slow process and needs to be implemented several weeks before surgery to gain maximal benefit. Anemia should be corrected, as far as possible, preoperatively, as decreasing preoperative hemoglobin concentrations is associated with increased morbidity and mortality in such patients. Intraoperative measures include cell salvage of blood loss and acute normovolemic hemodilution to minimize red cell loss. Some patients will accept these techniques while others will refuse. We adopted the strategy of cell salvage technique in our case as well. Strategies to minimize postoperative blood loss include a prompt assessment by a senior clinician if acute blood loss is suspected, the rationalization of postoperative phlebotomy, and the use of pediatric tubes where available [7]. In certain surgical specialties, postoperative wound drainage systems have been designed to allow the autotransfusion of drained blood, minimizing any loss, although the evidence for this is contradictory [8]. 

A study comparing the outcomes of 31 Jehovah's Witness patients with a similar control group undergoing major cardiac surgeries showed similar results in terms of hospital stay and mortality in centers that practiced a rigorous blood product management protocol [9]. A debrief should also be conducted with the team at the end of the operation, and postoperative strategies should be documented clearly in the case notes. All personnel (including nursing and support staff) involved in the postoperative care of these patients should be made aware of the patient's wishes and the postoperative monitoring and treatment plan, as was done in our case [10]. The abstract of this article was presented at the 'American College of Cardiology Conference' in March 2019 [10]. 

## Conclusions

Complex ACHD surgery can be done in Jehovah's Witness patients with skilled perioperative and intraoperative management. The role of bovine hemoglobin in this population is being defined and was found helpful in this case. Here, we describe a successful pulmonary valve replacement (PVR) and left pulmonary artery (PA) augmentation in an adult Jehovah's Witness patient with prior tetralogy of Fallot repair and velocardiofacial syndrome. A multimodal strategy to optimize the intraoperative and perioperative blood loss usually makes complex surgeries, including complex cardiac surgeries in adult Jehovah's Witness patients, possible with favorable outcomes.
